# The Spectrum of Beta-thalassemia Mutations in Couples Referred for Chorionic Villus Sampling at Bahawal Victoria Hospital, Bahawalpur

**DOI:** 10.7759/cureus.3265

**Published:** 2018-09-07

**Authors:** Uffan Zafar, Kamran Naseem, Muhammad Usman Baig, Zain Ali Khan, Fariha Zafar, Saba Akram

**Affiliations:** 1 Radiology Department, Bahawal Victoria Hospital, Quaid-E-Azam Medical College, Bahawalpur, PAK; 2 Radiology Department, Bahawal Victoria Hospital, Quaid-E-Azam Medical College, Bahawalpur , PAK; 3 Medicine, Bahawal Victoria Hospital, Bahawalpur, PAK; 4 Gastroenterology, Pakistan Kidney and Liver Institute and Research Centre, Okara, PAK; 5 Community Medicine, Quaid E Azam Medical College, Bahawalpur, PAK; 6 Medical Ward, Bahawal Victoria Hospital, Quaid-E-Azam Medical College, Bahawalpur, PAK

**Keywords:** chorionic villus sampling, beta thalassemia, spectrum of mutations, prenatal diagnosis

## Abstract

Introduction

The prevalence of beta-thalassemia mutations is different in various castes, regions, and ethnic groups. By knowing this prevalence, we can conduct a targeted screening of only the high-risk population and only for the specific mutations that are prevalent in each group.

Objective

The purpose of this study was to determine the regional, caste-wise, and ethnic spectrum of beta-thalassemia mutations in couples referred for a prenatal diagnosis.

Methods

A cross-sectional analytical study was conducted at the thalassemia unit, Bahawal Victoria Hospital, Bahawalpur, from October 1, 2015, to May 15, 2018. After obtaining informed consent, chorionic villus sampling (CVS) was performed in 144 women having a gestational age of 12 to 16 weeks. We took blood samples of the couples. A chromosomal analysis for 13 mutations was done at Punjab Thalassaemia Prevention Programme (PTPP), Lahore. The researchers filled a questionnaire with all the details of couples like ethnicity, caste, and region.

Results

The most common mutation was Fr 8-9(+G), accounting for 29.8%, followed by IVS 1-5(G-C), which was 28.9%. We did not find three mutations in any chromosome. Fr 8-9 (+G) was the most common mutation among Punjabis and Pakhtoons. IVS 1-5 (G-C) was the most common mutation among Saraikis and Urdu-speaking people. In Rajputs, Arains, Jatts, and Pathans, Fr 8-9 (+G) was the most common mutation. IVS 1-5 (G-C) was the most common mutation among Sheikhs, Balochs, Syeds, and Miscellaneous. IVS 1-5 (G-C) was the most common mutation in the Bahawalpur division and Ghotki (Sindh) while Fr 8-9 (+G) was the most common mutation in the Multan division. The p-value of all the results was <0.001.

Conclusion

There is an ethnic, caste-wise, and regional distribution of mutations. We can conduct a targeted screening of the population and provide counseling about chorionic villus sampling by using this local data.

## Introduction

Beta thalassemia is a hemoglobin disorder that is one of the most common, single-gene, inherited disorders [1]. It severely affects the quality of life of patients and their parents [2]. The annual incidence of symptomatic beta thalassemia is nearly one in 100,000 in the whole world [3]. There are almost eight to 9.8-million carriers in Pakistan. The incidence of beta thalassemia in Pakistan is estimated to be 5000-9000 live births per year [4]. The culture of marrying within the boundaries of caste, ethnicity, and consanguinity entraps the pool of mutations, which further increases the incidence of beta thalassemia [5].

Patients with thalassemia major require an expensive bone marrow transplant. So, prevention of beta thalassemia is always far better than cure. The three stages approach developed for prevention is premarital carrier screening, limiting the family size of the parents with an affected child, and prenatal diagnosis by chorionic villus sampling (CVS) [6]. The screening program will be efficient in regions and ethnic groups with a higher prevalence of mutations. Moreover, we cannot do the screening of more than 300 mutations together in the target population. We should screen just those mutations that are more prevalent in specific ethnicities and regions. To do this targeted screening, we have to determine the regional, ethnic, and caste-wise distribution of mutations. In this way, we can make a cost-effective and comprehensive strategy for carrier detection and thus for thalassemia prevention [7].

Bahawalpur is the largest division of Punjab, by area, with a population of more than 11 million. There is a cultural leaning towards marrying within the same caste. Consanguineous marriages are also common. We could not find any study on the spectrum of beta-thalassemia mutations in Bahawalpur in the last 11 years. There is no published data on CVS performed in these regions to help in counseling couples about the prenatal diagnosis and termination of pregnancy in case of beta-thalassemia major. This study will provide data to start educational programs that can significantly reduce thalassemia major. Moreover, it will help form diagnostic kits with only the common mutations to reduce the cost of screening and prevention programs. The study will determine the regional, caste-wise, and ethnic spectrum of beta-thalassemia mutations in the couples (and their fetus) referred for prenatal diagnosis, thereby helping in forming a targeted approach for the screening of high-risk people.

## Materials and methods

It was a cross-sectional analytical study. The study place was the thalassemia unit, Bahawal Victoria Hospital, Bahawalpur. It was performed from October 1, 2015, to May 15, 2018. We included 144 couples having 576 chromosomes and 144 samples of CVS having 288 chromosomes in the study. A total of 864 chromosomes were analyzed. The non-probability convenient sampling technique was used. The study was approved by the institutional ethical review committee (Department of Medical Education) of Quaid e Azam Medical College, Bahawalpur. We took written informed consent from the couples. Parents having one or more children with beta-thalassemia major and both parents having beta-thalassemia minor were included. Pregnant mothers with a gestational age of 12-16 weeks were included. Collected data included details of the caste, ethnicity, and region of the members. We excluded couples with tuberculosis (TB), cardiovascular, renal, and coagulation disorders, and other major health problems from the study. The researchers filled a questionnaire with all the details of couples like ethnicity, caste, and region through face to face interviews. Chorionic villus sampling was performed during the gestational age of 12-16 weeks. After proper counseling and consent, the placental sample was drawn through the transabdominal approach. We collected blood samples of the couples in ethylenediaminetetraacetic acid (EDTA) coated vials. Deoxyribonucleic (DNA) extraction was done by using the Genomic DNA Purification Kit (Gentra Systems, Minnesota, USA). Amplification Refractory Mutation System - Polymerase Chain Reaction (ARMS-PCR) was used for a genetic analysis of 13 mutations, namely, Fr 8-9(+G ), IVS 1-5(G-C), Fr 41-42(-TCTT), Cd-15 (G-A,), Cd-5 (-CT), IVS 1-1(G-T), IVS 1-1(G-A), Cd-30(G-C), Cd-30 (G-A), Fr 16 (-C), IVS II-1 (G-A), Del 619 bp, and Cap+ 1 (A-C). While analyzing data, only mutated alleles were analyzed. For data analysis, we used SPSS 16 (SPSS Inc., Chicago, IL, US).

## Results

We studied 144 couples and 144 samples of chorionic villus sampling. These consisted of a total of 864 chromosomes. They belonged to Bahawalpur division, three districts of Multan division (District Vehari, District Lodhran, District Multan), and District Ghotki from Sindh province. Seventy-five percent couples from Bahawalpur division, 23.6% couples from Multan division, and 1.4% couples from Ghotki Sindh enrolled in our study. We found five ethnic groups to be prevalent in our study. Saraikis (81.2%) were the most common, followed by Punjabis(13.9%), Urdu speakers (2.1%), Sindhis (1.4%), and Pakhtoons(1.4%). Caste-wise, Rajputs (32.6%) were the most common followed by Jatts (20.8%), Balochs (18.1%), Arains (8.3%), Sheikhs (3.5%), Pathans (2.8%), Syeds (2.1%), and Miscellaneous (11.8%).

**Figure 1:**
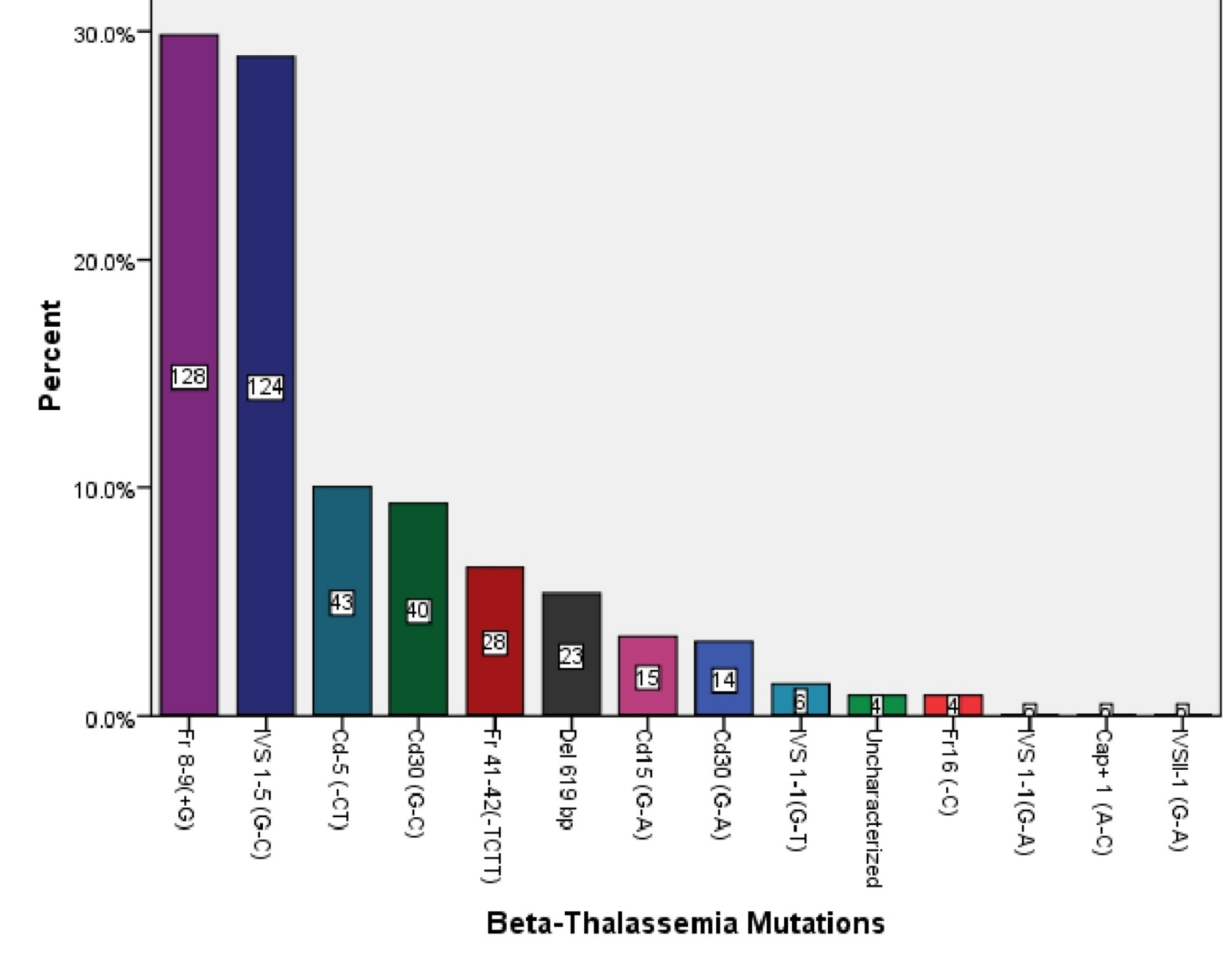
Percentage Frequency of Different Beta-Thalassemia Mutations

All samples had a total of 429 mutations. The most common was Fr 8-9(+G) followed by IVS 1-5 (G-C). We could not find IVS 1-1 (G-A), IVS II-1 (G-A), and Cap+ 1 (A-C) in any chromosome. Five mutations constituted more than 80%. Figure 1 shows the percentage and count of all the mutations found in our study.

**Figure 2:**
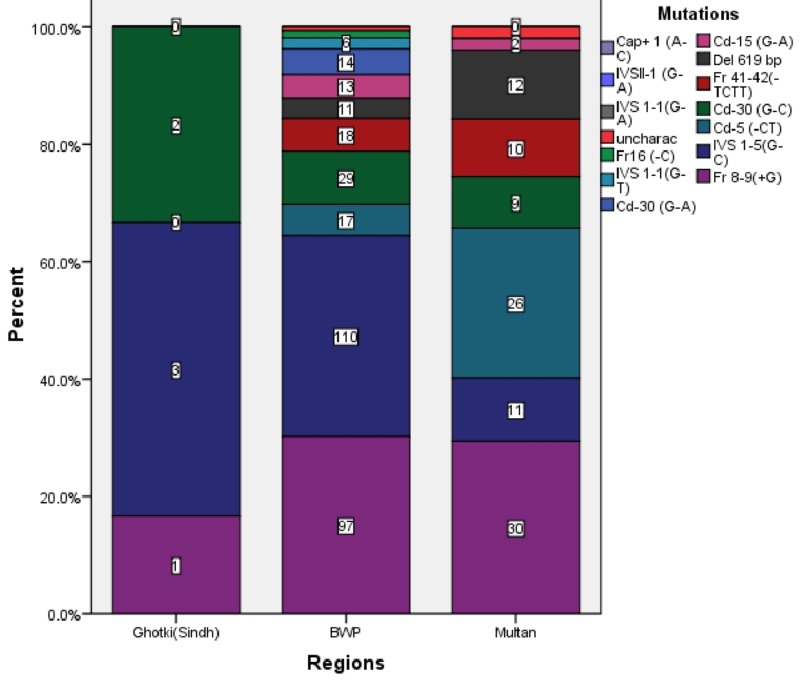
Regional Distribution of Beta-Thalassemia Mutations

The p-value of all the results in our study was <0.001. The spectrum of beta-thalassemia mutations was different in different divisions and even in different districts of the same division. IVS 1-5 (G-C) was the most common mutation in Bahawalpur division and Ghotki (Sindh) while Fr 8-9 (+G) was the most common mutation in Multan division. The district-wise analysis showed that all the districts of Bahawalpur division had Fr 8-9(+G) as the most common mutation except Bahawalpur district, which had IVS 1-5(G-C). In Multan division, District Multan and District Vehari had Fr 8-9(+G) but District Lodharan had cd-5 (-CT) as the most common mutation. Figure 2 shows the regional distribution of mutations.

**Figure 3:**
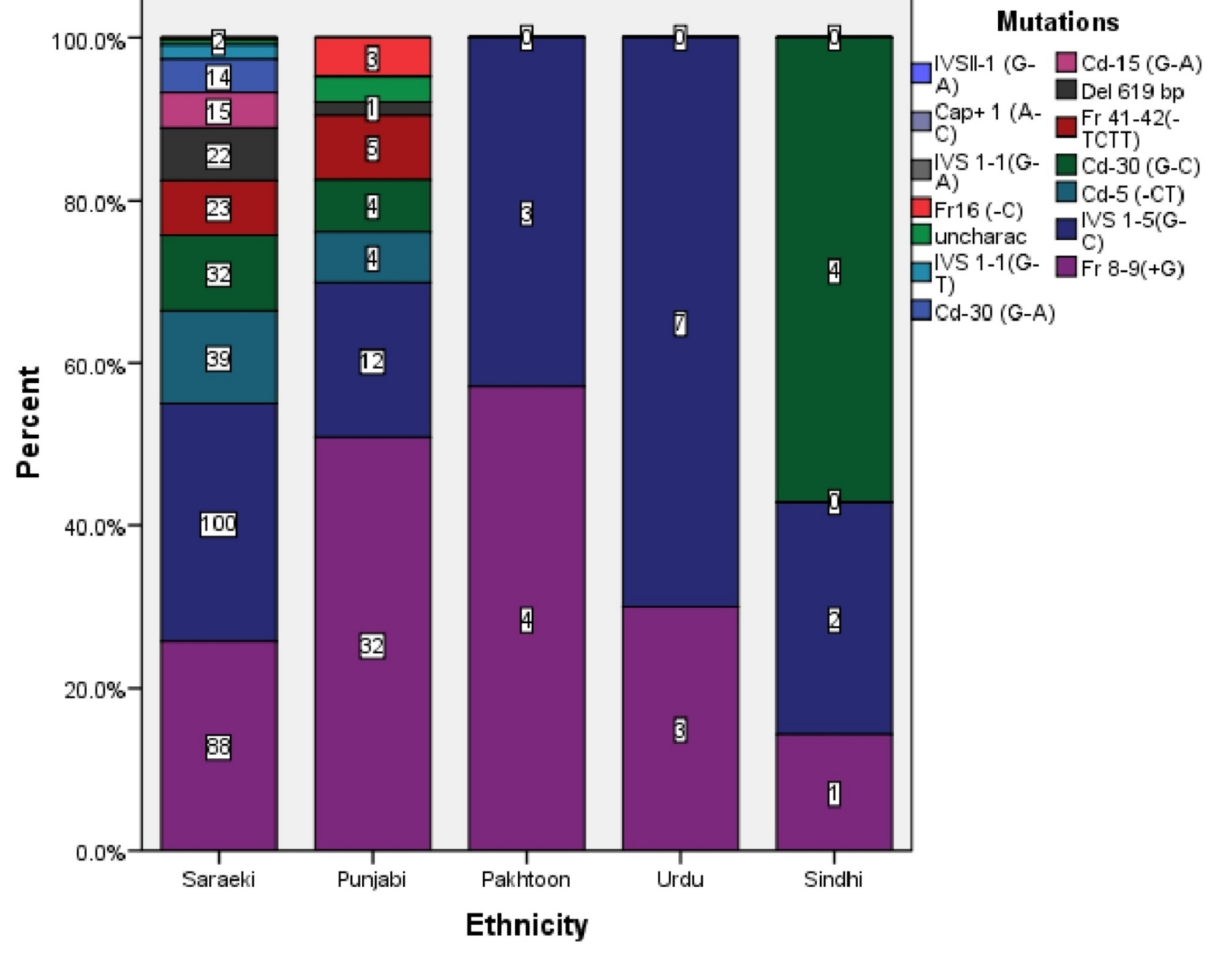
Ethnic Distribution of Beta-Thalassemia Mutations

Regarding ethnicity, we found that Saraikis (79.7%) had the maximum number of mutations followed by Punjabis (14.7%), Urdu speakers (2.33%), Pakhtoons (1.63%), and Sindhis (1.63%). Fr 8-9 (+G) was the most common mutation in Punjabis and Pakhtoons. IVS 1-5 (G-C) was the most common mutation among Saraikis and Urdu speakers. Figure 3 demonstrates the distribution of mutations in each ethnic group.

**Figure 4:**
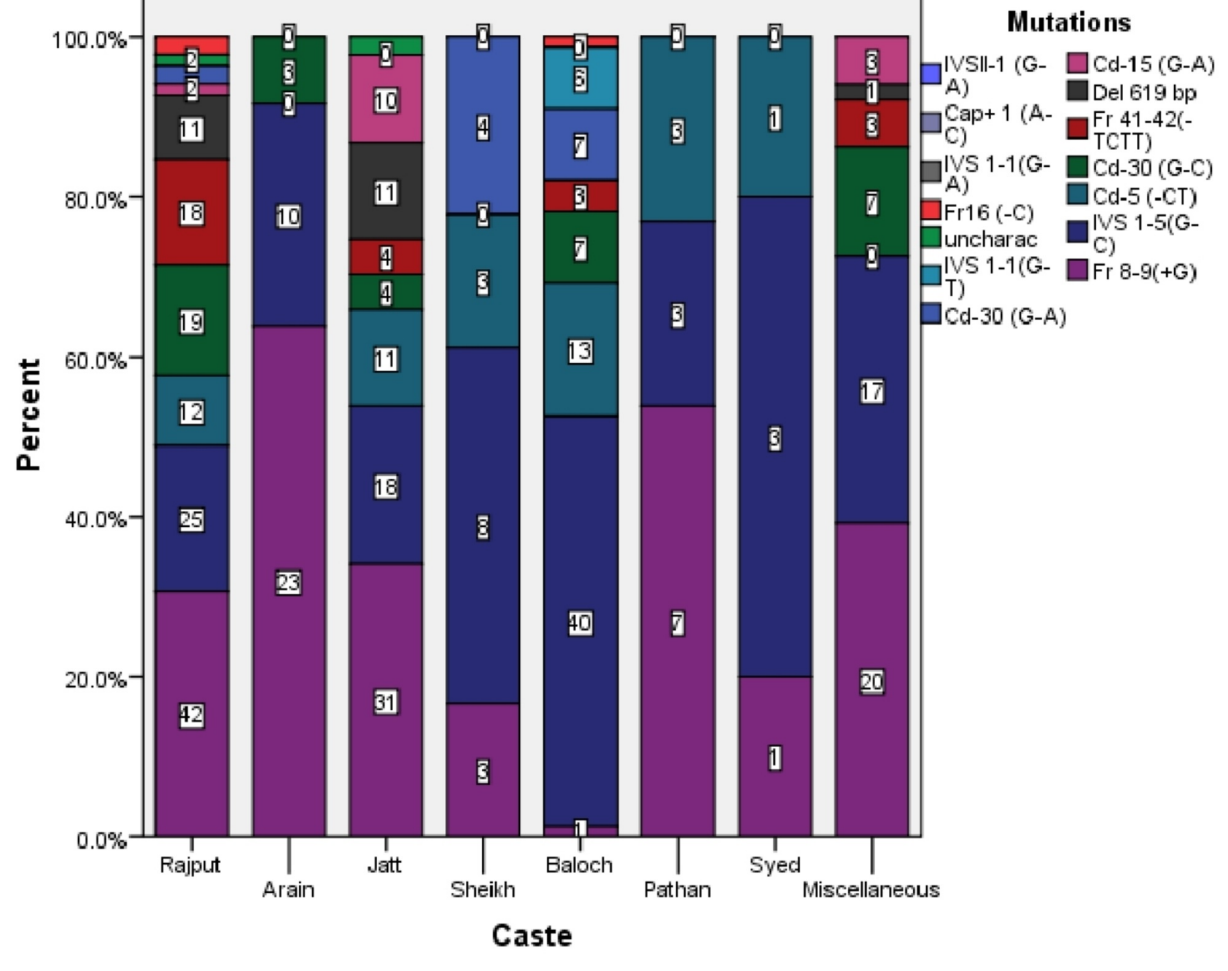
Caste-wise Distribution of Beta-Thalassemia Mutations

Caste-wise, the mutations were more prevalent in Rajputs (31.9%) followed by Jatts (21.2%), Balochs (18.2%), Arains (8.4%), Sheikhs (4.2%), Pathans (3%), Syeds (1.1%), and Miscellaneous (11.9%). Figure 4 shows Fr 8-9 (+G) as the most common mutation in Rajputs, Arains, Jatts, Pathans, and Miscellaneous castes but IVS 1-5 (G-C) is the most common mutation in Sheikhs, Balochs, and Syeds.

## Discussion

We found that the spectrum of beta-thalassemia mutations was different in various regions, castes, and ethnicities. Overall, Fr-8-9(+G) was the most common mutation (30.1%) followed by IVS 1-5(G-C), which was 29.2%. We performed our study in divisions of South Punjab. In another study conducted 12 years ago in South Punjab, IVS 1-5(G-C) was the most common mutation (42%) followed by Fr 8-9(+G), which was 40% [8]. This difference is probably due to the shift in the frequencies of mutations. Studies in India recommended an evaluation of the spectrum of beta-thalassemia mutations on a regular basis due to the high shift in frequencies of mutations [5,9]. IVS 1-1 (G-A), IVS II-1 (G-A), and Cap+ 1 (A-C) were not present in our study participants. In another study, these three mutations were present in all the divisions of Punjab except Dera Ghazi Khan division (the only division of South Punjab not included in our study) [8]. So, we can exclude these mutations from diagnostic kits of this region to make this diagnosis and screening process cost-effective.

In Punjabi speakers, Fr 8-9(+G) was the most common mutation, which is the same as in a study carried out in the Punjabi-speaking region of Punjab [10]. We found that Pakhtoons had a higher prevalence of Fr 8-9(+G), which is the same as in Pakhtoons in a study carried out in Karachi [7]. In the Saraiki and Urdu-speaking communities, IVS 1-5(G-C) was the most common mutation. It is similar to a study conducted in the Saraiki-predominant division Dera Ghazi Khan and to another in the Urdu-speaking community of Karachi [8,11]. The Urdu-speaking community migrated from India. They had IVS 1-5(G-C) as the most common mutation, which is the same as the most common mutation seen in a study in India [9].

In Bahawalpur division as a whole, IVS 1-5(G-C) was the most common mutation (34.2%) followed by Fr 8-9(+G) (30.2%). All the districts of Bahawalpur division had Fr 8-9(+G) as the most common mutation except Bahawalpur district, which had IVS 1-5(G-C). Similarly, in Multan division, Fr 8-9(+G) (29.4% or 30/102) was the most common mutation. All the regions of Multan division included in our study had Fr 8-9(+G) as the most common except Lodharan district, which had cd5 (-CT). We found IVS 1-5(G-C) as the most common in Ghotki (Sindh). Another study shows that IVS 1-5(G-C) is the most common mutation in Karachi (Sindh) [11]. Sindh shares a border with India. It has IVS 1-5(G-C), which is the same as the most common mutation described in a study conducted in India [9].

Caste-wise, the mutations were more prevalent in Rajputs (31.9%), followed by Jatts (21.2%), Balochs (18.2%), Arains (8.4%), Sheikhs (4.2%), Pathans (3%), Syeds (1.1%), and Miscellaneous (11.9%). It is comparable to another study in Punjab except that Balochs had a higher percentage of mutations in our study [12]. It is probably due to a larger number of Baloch people residing in South Punjab due to its proximity to the Balochistan province. Fr 8-9(+G) was the most common mutation in Rajputs, Arains, Jatts, and Pathans. In Sheikhs, Balochs, and Syeds, IVS 1-5(G-C) was the most common mutation.

Regarding ethnicity, we found that Saraikis had the maximum number of mutations (79.7%), followed by Punjabis (14.7%), Urdu speakers (2.33%), Pakhtoons (1.63%), and Sindhis (1.63%). In a study conducted in the Punjabi-speaking predominant region of Pakistan, beta thalassemia was prevalent in Punjabis (60.7%), followed by Saraikis (25.5%) [12].

Knowledge about the regional, ethnic, and caste-wise distribution of mutations helps effectively target the people at risk at all the levels of prevention. We can prevent beta thalassemia by targeted carrier detection, genetic counseling, and prenatal diagnosis. World Health Organization (WHO) also recommended an efficient approach for the prevention of beta thalassemia by documenting the molecular heterogeneity of beta-thalassemia mutations [6]. The shift in the spectrum of the beta-thalassemia mutations suggests that we should analyze this spectrum on a regular basis. In this way, we can find those mutations that are no more appearing in the population and remove their primers from the diagnostic kit. It makes a cost-effective and targeted approach for thalassemia detection. Our study has limitations in the sense that we have not compared the demographic characteristics of the study participants with the general population while analyzing the data.

## Conclusions

There is regional, ethnic, and caste-wise heterogeneity of beta-thalassemia mutations in the varied population of South Punjab. The local data will be used to counsel about chorionic villus sampling and pregnancy termination in case of a fetus with beta-thalassemia major. It will reduce the cost of screening and provide data about high-risk communities to effectively target them for genetic counseling, pre-marital carrier screening, and then prenatal diagnosis after marriage. All these measures will decrease the incidence of beta thalassemia.
